# Flavonoid biosynthesis controls fiber color in naturally colored cotton

**DOI:** 10.7717/peerj.4537

**Published:** 2018-04-18

**Authors:** Hai-Feng Liu, Cheng Luo, Wu Song, Haitao Shen, Guoliang Li, Zhi-Gang He, Wen-Gang Chen, Yan-Yan Cao, Fang Huang, Shou-Wu Tang, Ping Hong, En-Feng Zhao, Jianbo Zhu, Dajun He, Shaoming Wang, Guang-Ying Huo, Hailiang Liu

**Affiliations:** 1 Laboratory of Agricultural Biotechnology, College of Life Science, Shihezi University, Shihezi, Xinjiang, China; 2 China Colored-cotton (Group) Co., Ltd., Urumqi, Xinjiang, China; 3 National Key Laboratory of Crop Genetic Improvement, Hubei Key Laboratory of Agricultural Bioinformatics, College of Informatics, Huazhong Agricultural University, Wuhan, China; 4 Translational Stem Cell Research Center, Tongji Hospital, School of Medicine, Tongji University, Shanghai, China

**Keywords:** Fiber color, Naturally colored cotton, Flavonoid biosynthesis, Fiber quality, Transcriptome analysis, RNA interference

## Abstract

The existence of only natural brown and green cotton fibers (BCF and GCF, respectively), as well as poor fiber quality, limits the use of naturally colored cotton (*Gossypium hirsutum* L.). A better understanding of fiber pigment regulation is needed to surmount these obstacles. In this work, transcriptome analysis and quantitative reverse transcription PCR revealed that 13 and 9 phenylpropanoid (metabolic) pathway genes were enriched during pigment synthesis, while the differential expression of phenylpropanoid (metabolic) and flavonoid metabolic pathway genes occurred among BCF, GCF, and white cotton fibers (WCF). Silencing the chalcone flavanone isomerase gene in a BCF line resulted in three fiber phenotypes among offspring of the RNAi lines: BCF, almost WCF, and GCF. The lines with almost WCF suppressed chalcone flavanone isomerase, while the lines with GCF highly expressed the glucosyl transferase (*3GT*) gene. Overexpression of the * Gh3GT* or *Arabidopsis thaliana 3GT* gene in BCF lines resulted in GCF. Additionally, the phenylpropanoid and flavonoid metabolites of BCF and GCF were significantly higher than those of WCF as assessed by a metabolomics analysis. Thus, the flavonoid biosynthetic pathway controls both brown and green pigmentation processes. Like natural colored fibers, the transgenic colored fibers were weaker and shorter than WCF. This study shows the potential of flavonoid pathway modifications to alter cotton fibers’ color and quality.

## Introduction

Cotton is the largest natural textile material, which is accounting for a large proportion of economy. The increasing use of naturally colored cotton reflects the consumers’ desire to use natural products. Based on fiber color, there are two basic types of cotton, namely brown and green (Du, Zhang & Yuan, 1997). However, the fiber quality of naturally colored cotton is poor, which largely limits the use and development of colored cotton. Lots of effects have been applied to improve the quality of naturally colored cotton, while the results are not good as expected. Traditional breeding methods cannot produce new cultivars of naturally colored cotton because of the lack of germplasm for different colored cottons. Therefore, biotechnology for producing new cultivars of naturally colored cotton is required to address this issue.

The cotton fiber color has been studied in many studies. Previous studies show, that the pigmentations of brown cotton fibers (BCF) and green cotton fibers (GCF) might be affected by the flavonoid biosynthetic pathway (Zhang et al., 2011; Li et al., 2012; Hua et al., 2007; Xiao et al., 2007; Li et al., 2013; Feng et al., 2013; Tan et al., 2013; Xiao et al., 2014). Flavonoids form the largest class of naturally occurring secondary metabolites, with the majority being colored. They are the main components of plant pigments (Winkel-Shirley, 2001; Grayer & Harborne, 1994; Forkmann & Martens, 2001) and have been studied in many plants, such as *Arabidopsis thaliana* and *Petunia hybrida* (Buer & Muday, 2004). However, the knowledge is limited in the application of the flavonoid biosynthetic pathway to influence cotton fiber pigment development.

The objectives of this study were to characterize the genetic mechanisms regulating pigment formation in colored cotton fibers and explore the possibility of changing the color of cotton through biotechnological techniques. The methods used are RNA Sequencing, metabolome, RNAi and so on. RNA sequencing (RNA-Seq) is a technique with next-generation sequencing (NGS) to evaluate the presence and quantity of RNA in a biological sample, which is the basis and starting point of gene function and structure research in this research. Differentially-expressed genes from different-colored cottons with RNA-Seq were examined. Metabolomics is a bridge between genes and phenotypes, which can provide a new way for functional genomics research to efficiently and rapidly validate large-scale gene functions. Cultivars with transgenes from RNA-Seq and metabolome were used to confirm that the flavonoid biosynthesis genes not only controlled fiber color but also influenced fiber quality.

## Materials and Methods

### Plant materials

Brown cotton (*Gossypium hirsutum* L.) cultivars ‘Zong 1282’ and ‘Xincaimian 5’, and their near-isogenic lines with white cotton fibers (WCF) were grown in two experimental fields at Korla (Xinjiang, China) and Danzhou (Hainan, China) using standard agronomic conditions and practices. Ovules and fibers were collected 0 and 12 d post-anthesis (DPA) and stored at −80 °C before use.

### RNA sequencing, mapping, and transcript assembly

In total, 12 libraries were sequenced (samples: BCF, GCF, and WCF, numbered 5, 6, and 24, respectively at two time points, 0 and 12 DPA) using the HiSeq 2000 Sequencing System (Illumina, San Diego, CA, USA) at the Genome Center of WuXi App Tec (Shanghai, China). The raw reads were evaluated with software FsatQC (v0.10.1) for quality control and were trimmed with software Trimmomatic (v0.32) by removing adaptors and low-quality reads to generate clean reads. The clean reads were aligned with the *Gossypium raimondii* genome sequence (G.raimondii_JGI_221_v2.1) (Wang et al., 2012) using the spliced read aligner Tophat (version 2.0.9) with default parameters. The genome index was generated by bowtie (v2.2.4) (Langmead & Salzberg, 2012). The transcripts were reconstructed using Cufflinks (version 2.2.0) that were annotated in Ensemble, and the transcript expression levels were estimated using reads per kilobase per million mapped reads (Kim et al., 2013). To eliminate poorly reconstructed transcripts, alignment artifacts, and background expression, transcripts with a mean coverage below one read per base were removed from the transcriptome. All the mapped reads were count by HTSeq (0.6.0) (Anders, Pyl & Huber, 2015). DESeq (1.18.0) (Anders & Huber, 2010) was used for differential expression analysis and genes with adjusted *p*-value less than 0.05 were considered as differentially expressed genes (DEGs).

### Quantitative reverse transcription PCR (qRT-PCR)

Total RNA was extracted according to a published method involving guanidine thiocyanate (Liu et al., 2006) and RNA samples were treated with Ambio™ DNaseI (Invitrogen, Carlsbad, CA, USA) and reverse transcribed with oligo (dT) primers (Invitrogen, Carlsbad, CA, USA). The qRT-PCR assay was performed using an ABI 7900HT Real-Time PCR System (Applied Biosystems, Foster City, CA, USA) with the QuantiTect SYBR Green PCR kit (Qiagen, Hilden, Germany). The PCR program was as follows: 2 min at 50 °C; 2 min at 95 °C; 40 cycles of 15 s at 95 °C, and 60 s at 60 °C. This was followed by a standard dissociation protocol to ensure that each amplicon was a single product. All data were normalized against *UBQ 7* levels (Shi et al., 2006). We performed the qRT-PCR assay in triplicate for each of the three independent samples.

### Construct development

An RNA interference (RNAi) construct was generated using the pANDA-35HK vector with CaMV35S promoter and neomycin phosphotransferase II (*NPTII*) and hygromycin phosphotransferase (*HPT*) resistance genes to down-regulate the cotton chalcone flavanone isomerase gene *GhCHI-1*’s expression in BCF (Miki, Itoh & Shimamoto, 2005). A 224-bp *GhCHI-1* cDNA fragment was amplified by PCR (primers: 5′-CACCTGATTTTGAGAAGTTCATACGGGTG-3′ and 5′-AGTCAAGGAACCTT- GGCCTGAAATA-3′) to facilitate directional cloning into the Gateway pENTR vector. The recombinant plasmid was digested with restriction enzyme PvuI and directionally inserted into the RNAi pANDA vector using LR Clonase according to the manufacturer’s instructions (Invitrogen, Carlsbad, CA, USA). Overexpression constructs were also produced using the pANDA vector to independently up-regulate the cotton or Arabidopsis glucosyl transferase (*3GT*) genes’ expression level in BCF. The *At3GT* and *Gh3GT* coding sequences with independently introduced using XbaI and EcoRI, and XbaI and SacI, restriction enzyme sites, respectively, and were amplified by PCR (primers: 5′-GCTCTAGAGCATGACCAAACCCTCCGACC-3′ and 5′-GGAATTCCTCAAATAATGTTTACAACTGCATCC-3′, and primers: 5′-GCTCTAGAGC ATGGAAGGCTACAAGAATGCTT-3′ and 5′-CGAGCTCGTCAA- CTTGGTGGTAAGTGTTG-3′, respectively) to facilitate directional cloning into the pANDA vector, which was digested with the same restriction enzymes.

### Agrobacterium-mediated transformation of cotton through pistils

Just prior to anthesis, the flowers that were about to open were tied up using string and unopened petals were clamped to avoid cross-pollination. The cotton pistils were sprayed two or three times with a sucrose inoculation solution containing *Agrobacterium tumefaciens* GV3101 transformed with the pANDA vector, a binary vector carrying the CaMV35S:*GhCHI-1* hairpin, CaMV35S:*Gh3GT* or CaMV35S:*At3GT* construct in the afternoon of the first day of flowering using a modified pistil drip pollination method (Chen et al., 2010). After spraying *Agrobacterium* drops, the treated flowers were immediately clamped for 3 d to facilitate *Agrobacterium* transformation under humid and dark conditions.

### Genomic DNA extraction, PCR, and Southern blot analysis

Genomic DNA from the leaves of wild-type and transgenic cotton plants were isolated using a cetyltrimethylammonium bromide method (Paterson, Brubaker & Wendel, 1993). The positive transformants were identified using PCR amplifications of *NPTII* and *HPT* with gene-specific primers. The *NPTII* forward and reverse primer sequences were 5′-AGACAAGTTCCTCTTCGGGC-3′ and 5′-TGAAGATGAACAAAG- CCCTG-3′, respectively. The *HPT* forward and reverse primer sequences were 5′-AGGGCGAAGAATCTCGTGCT-3′ and 5′-AACCCGCTCGTCTGGCTAAG-3′, respectively. The expected sizes of the *NPTII* and *HPT* PCR products were 251 and 309 bp, respectively.

Positive transgenic plants were selected for Southern blot analysis. Genomic DNA (20 µg) was digested with EcoRI, separated on a 0.8% agarose gel, and blotted onto a Hybond-N+ nylon membrane (Amersham Biosciences, Buckinghamshire, UK). We used digoxigenin-labeled *NPTII* probes for hybridization, which was completed according to the DIG High Prime DNA Labeling and Detection Starter Kit I (Roche, Basel, Switzerland).

### The metabolomics cotton fiber analysis

BCF, GCF, and WCF were naturally dried for 30 d in a laboratory room under a constant temperature. Then, 1.0 g of sieved cotton was transferred to a glass vial (100 ml), 50 ml methanol was added and the samples underwent ultrasonic extraction for 1 h. After the hour, the supernatants were removed. Another 50 ml methanol was added, and the procedure was repeated two more times. The supernatants were combined and concentrated under 38 °C using a BUCHI Rotavapor R-100 (BUCHI Labortechnik AG, Flawil, Switzerland) and BUCHI Vacuum Pump V-100 (BUCHI Labortechnik AG). After concentration, the residue was further dissolved in methanol (25 ml). All samples were acquired by the LC-MS system following machine order. First, all chromatographic separations were performed using an ultra-performance liquid chromatography system (Waters, Milford, MA, USA). An ACQUITY UPLC BEH C18 column (100 mm × 2.1 mm, 1.7 μm; Waters) was used for the reversed phase separation. The column oven was maintained at 50 °C. The flow rate was 0.4 ml/min, and the mobile phase consisted of solvent A (water + 0.1% formic acid) and solvent B (acetonitrile + 0.1% formic acid). Gradient elution conditions were set as follows: 0–2 min, 100% phase A; 2–11 min, 0%–100% B; 11–13 min, 100% B; 13–15 min, 0%–100% A. The injection volume for each sample was 10 μl. A high-resolution tandem mass spectrometer SYNAPT G2 XS QTOF (Waters) was used to detect metabolites eluted from the column. The Q-TOF was operated in both positive and negative ion modes. For positive ion mode, the capillary and sampling cone voltages were set at 2 kV and 40 V, respectively. For negative ion mode, the capillary and sampling cone voltages were set at 0.5 k V and 40 V, respectively. The MS data were acquired in Centroid MSE mode. The TOF mass range was from 50 to 1,200 Da, and the scan time was 0.2 s. For the MS/MS detection, all precursors were fragmented using 20–40 eV, and the scan time was 0.2 s. During the acquisition, the LE signal was acquired every 3 s to calibrate the mass accuracy. Furthermore, to evaluate the stability of the LC-MS during the whole acquisition, a quality control sample (pool of all samples) was acquired after every 10 samples.

All data were processed using Progenesis QI (version 2.2) (Waters) for LC-MS data preprocessing. The pathway analyses of metabolites were performed with KEGG database sources (http://www.genome.jp/kegg/) to help identify the pathways that were most significantly altered.

### Fiber quality measurement

Fiber qualities, including fiber length (mm), strength (cN/tex), and micronaire were assessed using 15 g fiber for each sample (Green & Culp, 1990) and the Uster HVI 1000 fiber tester (Uster Technologies, Uster, Switzerland). Fiber length was measured as the upper half mean length (mm), and fiber strength was measured using a scale.

### Statistical analyses

All statistical analyses were completed using SPSS 13.0 software (SPSS, Chicago, IL, USA). The data were evaluated using a one-way ANOVA. Data were expressed as the means ± standard errors. ‘a’, ‘b’, ‘c’ means with different letters are significantly different at *P* < 0.05. Significant differences between results were based on *P* < 0.05, *P* < 0.01, and *P* < 0.001.

## Results and Discussion

### Flavonoid biosynthetic pathway genes are enriched during naturally colored cotton fiber development

**Figure 1:**
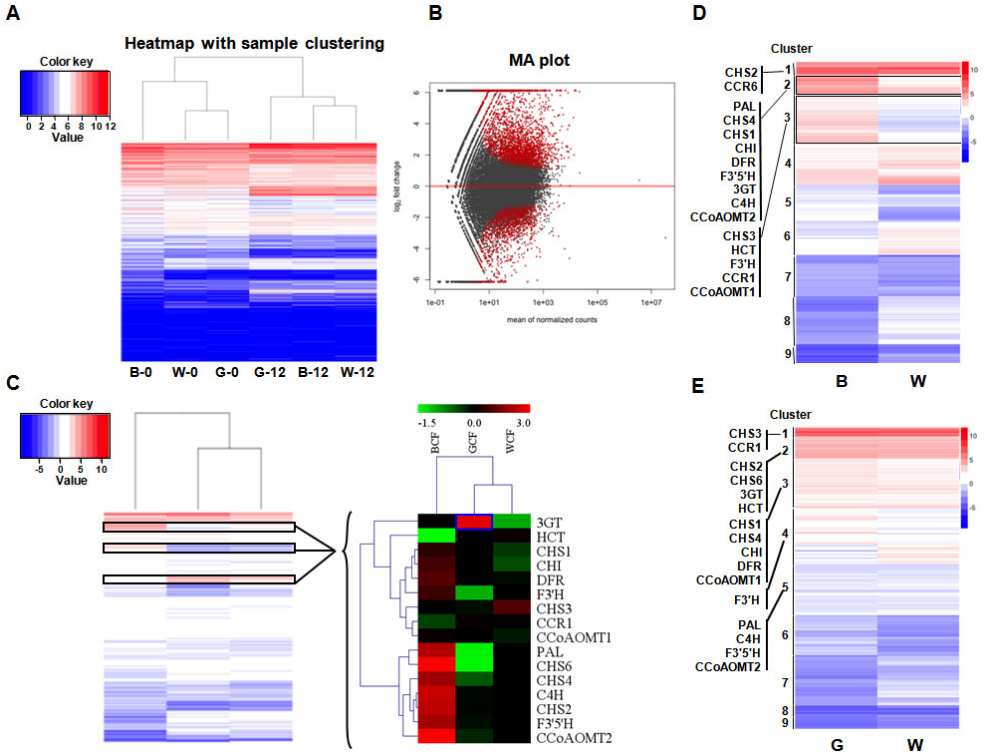
Transcriptome analysis of brown, green, and white fibers at 0 and 12 DPA. (A) Heatmap with sample clustering showing relative gene expression in six samples. B, brown cotton fiber; W, white cotton fiber; G, green cotton fiber; 0, 0 DPA; 12, 12 DPA. Relative expression levels are presented using a color scale ranging from saturated blue for log ratios ≤0, to saturated red for log ratios ≥12. Each gene is represented by a row of colored boxes. (B) MA plot visualization of the differentially expressed genes. DESeq2 comparisons are presented in the MA plots. We compared brown, green, and white fiber transcripts that were differentially expressed between 0 and 12 DPA. A *q*-value <0.1 was selected as the cutoff. Each dot represents a gene. Red dots represent significantly differentially expressed genes. (C) Hierarchical clustering analysis of the expression patterns of anthocyanin genes in brown, green, and white fibers. Relative expression levels are log_10_-transformed and presented using a color scale ranging from saturated blue for log ratios ≤−1.5, to saturated red for log ratios ≥3.0. Each gene is represented by a row of colored boxes. (D) Clustering of the transcripts that are differentially expressed in brown and white fibers between 0 and 12 DPA. Phenylpropanoid (metabolic) pathway genes were grouped into three clusters. (E) Clustering of the transcripts that were differentially expressed in green and white fibers between 0 and 12 DPA. Phenylpropanoid (metabolic) pathway genes were grouped into five clusters.

The expression profiles of BCF, GCF, and WCF at 0 and 12 DPA were assessed with RNA-Seq. There were 12 RNA-Seq libraries, each with 10.9–17.6 million raw reads separately. The mapped reads in each library were around 6.5–10.5 million reads. The genes with adjusted *p*-value less than 0.05 from DESeq were considered as differentially-expressed genes (refer to the ‘Materials and Methods’ section, and Table S1). Some genes involved in pigment synthesis were identified (Figs. 1A and 1B). In total, 13 and 9 genes associated with the phenylpropanoid (metabolic) pathway were up-regulated during pigment synthesis according to differential expression analyses in BCF and GCF, respectively (Fig. 1C). The expression levels were validated using qRT-PCR, which generated results that were consistent with the transcriptomics data (Fig. S1 and Table S2). The overall gene expression patterns were similar between GCF and WCF.

The expression levels of two key lignin biosynthesis enzymes in the phenylpropanoid (metabolic) pathway, *HCT* and *CCR*, were low in BCF. In contrast, the *3GT* gene was highly expressed in GCF. Genes related to the flavonoid metabolic pathway (Fig. S2 and Table S3), including *CHS* (*CHS1*; *CHS2*; *CHS4* and *CHS6*), *CHI*, *F*3′*H*, *F*3′5′*H*, *DFR*, and *3GT* were more highly expressed in BCF than in WCF (Fig. 1C).

In total, 16 genes belonging to the phenylpropanoid (metabolic) pathway that were differentially expressed in BCF and WCF were divided into Clusters 1, 2, or 3 (Fig. 1D and Table S4). A comparison between GCF and WCF indicated that the phenylpropanoid (metabolic) pathway genes were divided into five clusters (Fig. 1E and Table S5).

### RNAi-mediated inhibition of *GhCHI* in a BCF line resulted in three fiber phenotypes

**Figure 2:**
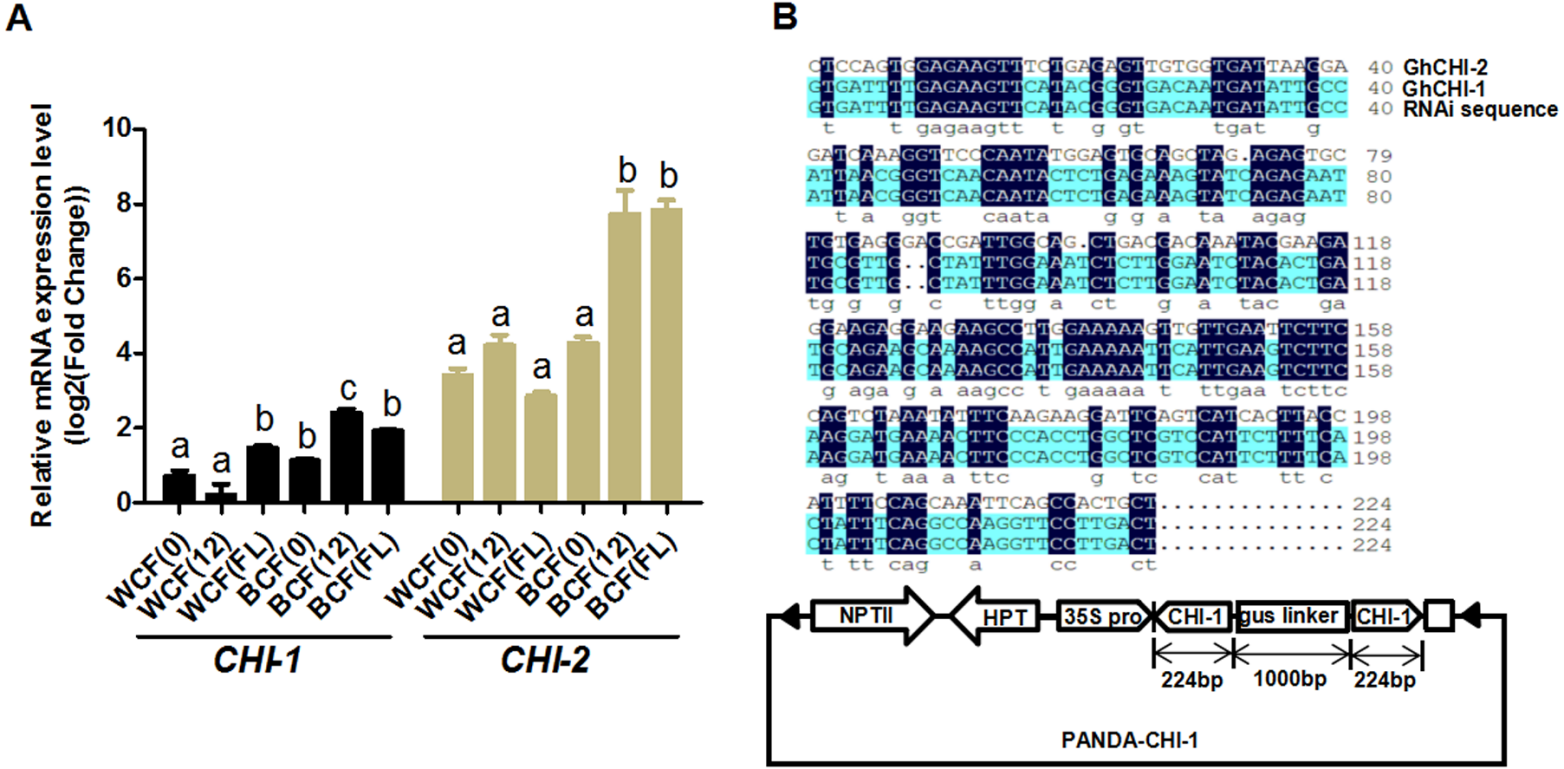
Analysis of *GhCHI-1* and *GhCHI-2* gene expression in brown cotton. (A) Expression analysis of * GhCHI-1* and *GhCHI-2* in brown cotton. 0, 0 DPA; 12, 12 DPA; FL, flower. Results are expressed as the means ± standard errors (*n* = 3). (B) Comparison of DNA sequences of *GhCHI-1*, *GhCHI-2*, and RNAi expression vector. The 224-bp *GhCHI-1* coding region was inserted into pANDA-35SHK to generate pANDA- *GhCHI-1*.

**Figure 3:**
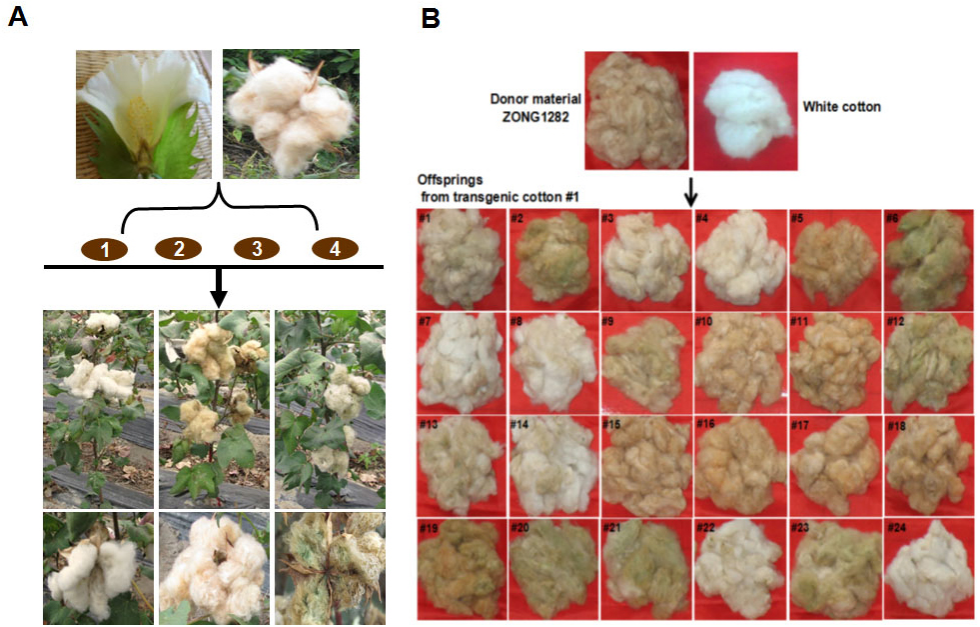
*Agrobacterium*-mediated transformation of brown cotton for RNAi-mediated inhibition of *GhCHI*. (A) *Agrobacterium*-mediated transformation of brown cotton (‘Zong 1282’). There were three main fiber color phenotypes in the *T*_1_ progeny of transgenic cotton. (B) Fiber color phenotypic analysis of 24 transgenic cotton #1 *T*_1_ progeny. Photo credit: Hai-Feng Liu.

**Figure 4:**
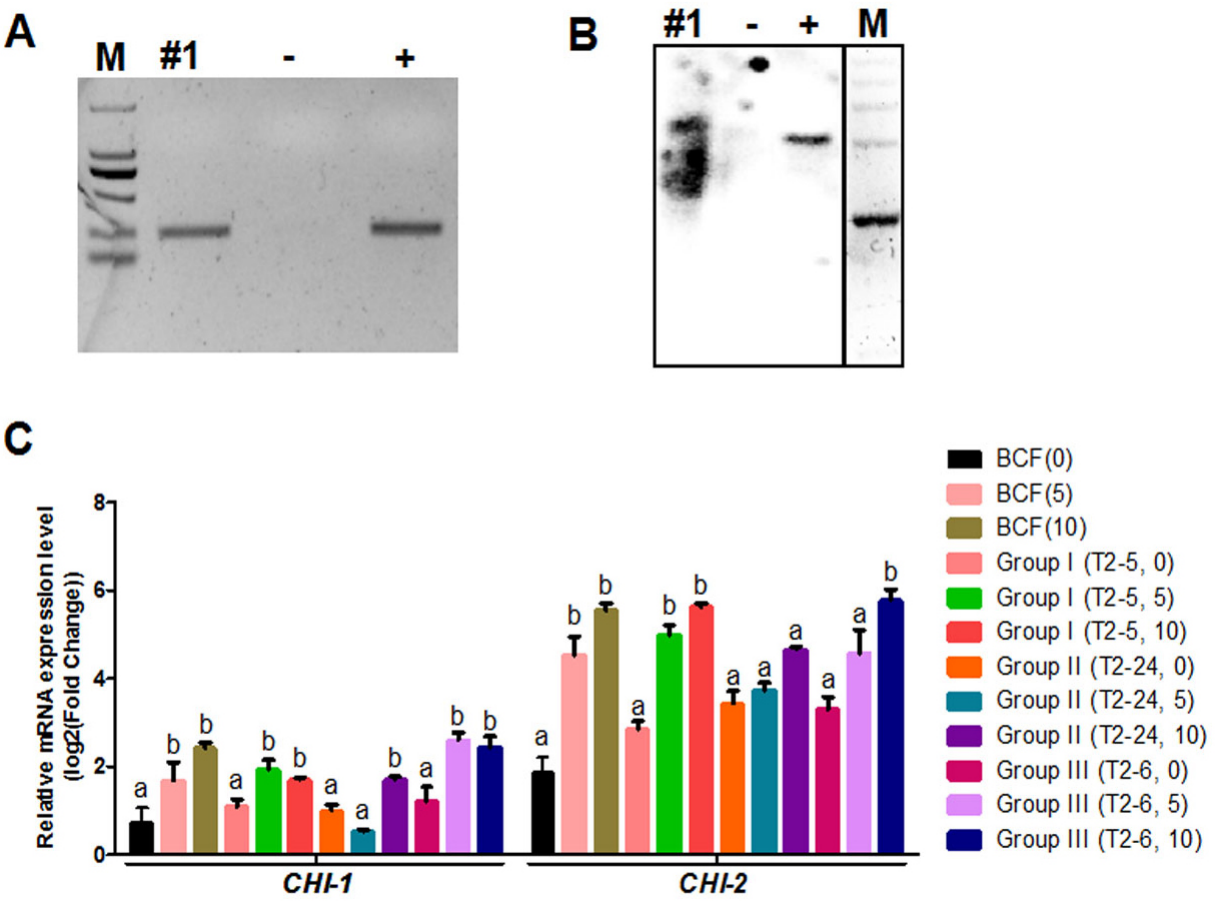
Identification of transgenic cotton and analysis of *GhCHI-1* and *GhCHI-2* gene expression. (A and B) PCR and Southern blot analysis of transgenic cotton #1 using primers specific for *NPTII*. M, DNA marker; +, pANDA vector positive control; −, non-transgenic cotton DNA negative control. (C) *GhCHI-1* and *GhCHI-2* expression analyses in *T*_1_ progeny of transgenic cotton #1. Results are expressed as the means ± standard errors (*n* = 3). ‘a’, ‘b’ , ‘c’ means with different letters are significantly different at *P* < 0.05 .

Whether the flavonoid biosynthetic pathway is responsible for the synthesis of brown pigment was examined by conducting RNAi experiments involving *CHI*, which is the second key gene in this pathway. Whether interfering with key flavonoid pathway genes could affect synthetic fiber pigment development. There are two *GhCHI* genes in upland cotton, *GhCHI-1* (gi|121755800|) and *GhCHI-2* (gi|295687228|), which are 29.26% identical at the amino acid level and 37.53% identical at the nucleic acid level (Fig. S3). They were expressed more highly at 12 DPA than at 0 DPA in BCF (Fig. 2A). A 224-bp fragment from the *GhCHI-1* gene’s coding region was inserted into the RNAi plant expression vector pANDA-35SHK to generate pANDA-*GhCHI-1* (Fig. 2B). Approximately 3,000 brown cotton plant (‘Zong 1282’; BCF) flowers were completed by using *Agrobacterium*-mediated transformation. Fibers of the original cotton cultivars did not undergo color changes, and they continued to grow after the harvest. We isolated four *T*_1_transgenic plants (transgenic cotton #1–4) from ∼3,000 originally transformed bolls, in which the fibers changed from brown to nearly white (Fig. 3A). These transgenic plants were analyzed by PCR amplifying the *NPTII* and *HPT* genes (Fig. 4A and Fig. S4). Southern blot analysis of T_1_ transgenic cotton #1 using an *NPTII*-specific probe confirmed the presence of three copies of the gene (Fig. 4B).

The three main fiber color phenotypes were observed in the T_1_ progeny (24 plants) of transgenic cotton #1 (cotton #2–4 expressed the same phenotype; Fig. S5) The T_1_ progeny plants were analyzed by PCR amplifying the *NPTII* gene (Fig. S6) and were categorized into three groups: Group I, unchanged BCF (T2–5, –10, –11, –15, –16, –17, –18, and –19); Group II, nearly WCF (T2–1, –3, –4, –7, –8, –13, –14, –22, and –24); and Group III, dark GCF (T2–2, –6, –9, –12, –20, –21, and –23) (Fig. 3B).

Expression analyses of 0-, 5-, and 10-DPA fibers from the transgenic plants of Groups I (T2–5), II (T2–24), and III (T2–6) revealed that *GhCHI-1* was suppressed in Group II at 5- and 10-DPA, but it was only significantly reduced at 10-DPA in Group I. At 5-DPA, the *GhCHI-1* expression level in Group I and Group III plants was higher than that in Group II plants. Additionally, the *GhCHI-1* expression level in Group I and Group III plants decreased from 5- to 10-DPA, but the *GhCHI-2* expression level was not affected. Only at 10-DPA was the *GhCHI-2* expression level in Group II plants was lower than in the BCF plants (Fig. 4C). However, *GhCHI-1* and *GhCHI-2* represent two copies of the *GhCHI* gene, with *GhCHI-2* being functionally redundant.

### Formation of green and brown pigments is controlled by the flavonoid biosynthetic pathway

**Figure 5:**
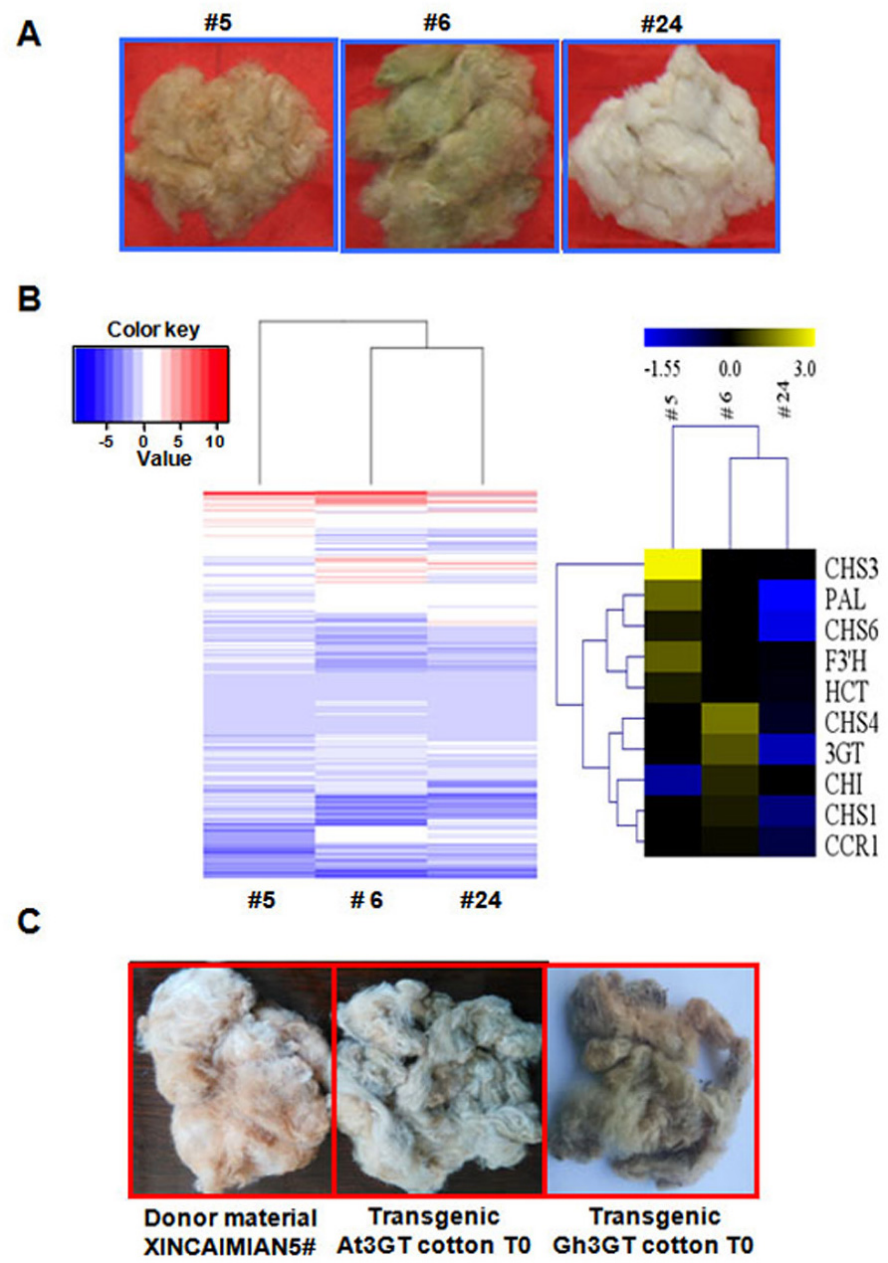
Transcriptome analysis of *T*_1_ progenies (#5, #6, and #24) originated from T0 transgenic cotton plant #1. (A) Three main fiber color phenotypes (#5, #6, and #24 corresponding to brown, dark green, and white, respectively). (B) Hierarchical clustering analysis of the expression patterns of anthocyanin genes in samples #5, #6, and #24. Relative expression levels were *lg*-transformed and presented using a color scale ranging from saturated blue for log ratios ≤−1.55, to saturated red for log ratios ≥3.0. Each gene is represented by a row of colored boxes. (C) Fiber color phenotypes of transgenic offspring overexpressing *Gh 3GT* and *At3GT*. The transgene was originated from ‘Xincaimian 5’ cotton. Photo credit: Hai-Feng Liu.

The differences in flavonoid biosynthesis existed were examined in the progeny of the transgenic plants in Groups I–III. The transcriptomes of fibers at 0- and 12-DPA were compared among Groups I (T2–5), II (T2–24), and III (T2–6) (Fig. 5A). The results were consistent with those described earlier. The differentially expressed GCF and WCF genes were clustered together. Seven differentially expressed genes were part of the phenylpropanoid (metabolic) pathway, including five genes involved in the flavonoid biosynthetic pathway. The level of gene expression in the flavonoid pathway was suppressed in the RNAi plants compared with the wild-type plants (Fig. 1C). *Gh3GT* was also still highly expressed in Group III (T2–6) *GhCHI-1* RNAi plants with green fibers (Fig. 5B and Tables S6 and S7).

Because *Gh3GT* is highly expressed in GCF, whether *Gh3GT* regulates pigment formation in GCF was examined. Two vectors were used to independently overexpress *Gh3GT* and *At3GT* (At5g17050) in ‘Xincaimian 5’ cotton (dark BCF). The overexpression of the *Gh3GT* gene resulted in GCF (Fig. 5C). The seedlings of the T1 generation of these transgenic plants were analyzed by PCR, amplifying the *NPTII* and (or) *HPT* genes (Fig. S4). The synthesis of the green pigment may result from the flavonoid biosynthetic pathway involving the 3GT enzyme. Subsequently, the BCF and GCF phenotypes, which are controlled by the flavonoid biosynthetic pathway, were confirmed using metabolomics to analyze BCF, GCF, and WCF (Table S8).

### Fiber quality correlated negatively with pigmentation in naturally colored cotton

**Figure 6:**
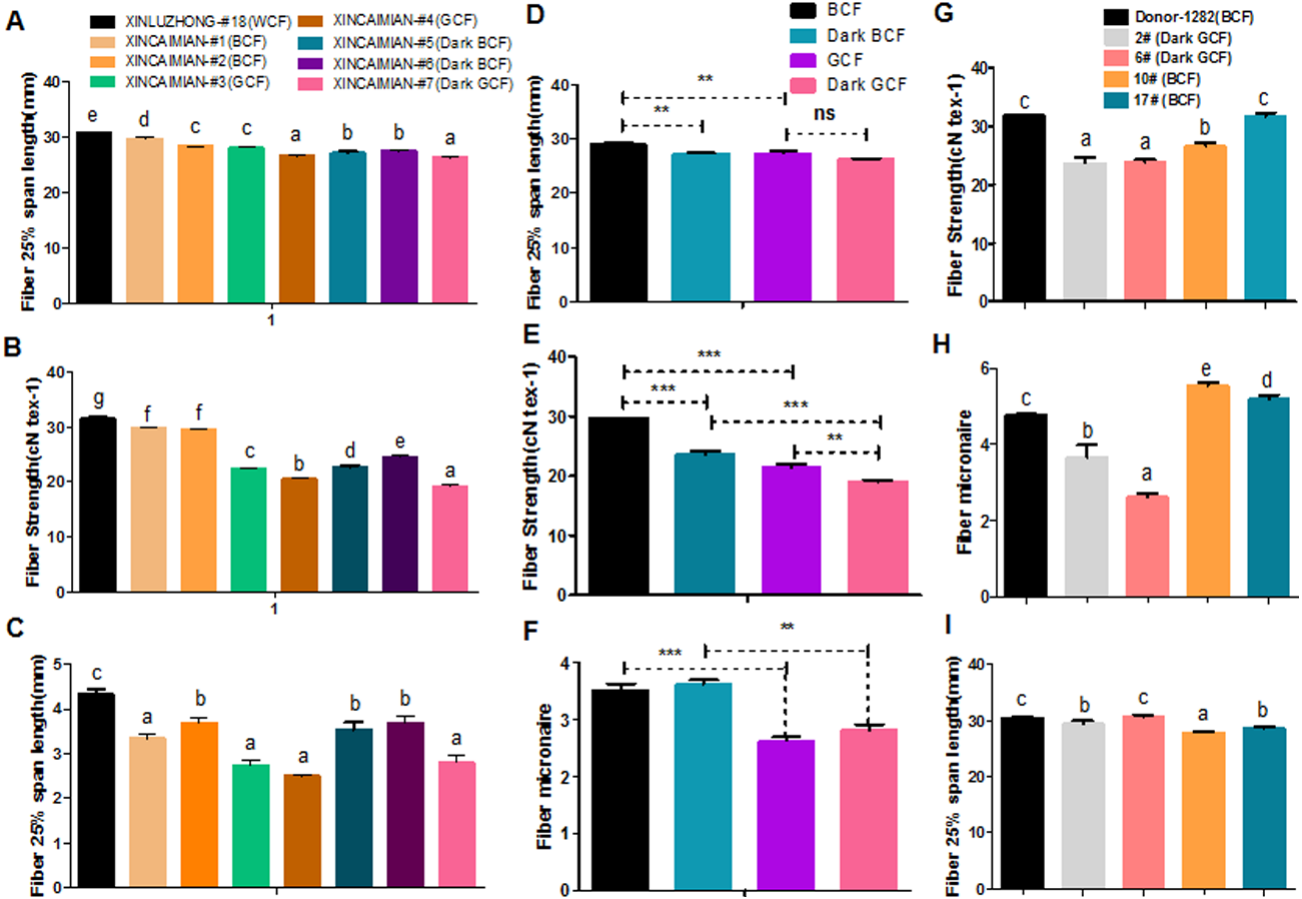
Correlation analysis of fiber color and quality. (A–C) Fiber quality comparison between naturally colored cotton and white cotton. ‘Xinluzhong’ sample #18: WCF. ‘Xincaimian’ samples #1, #2, #5, and #6: BCF. ‘Xincaimian’ samples #3 and #4: GCF. Results are expressed as the means ± standard errors (*n* = 3). (D–F) Fiber quality comparison between GCF and BCF. Results are expressed as the means ± standard errors (*n* = 3). (G–I) Fiber quality comparison among transgenic cotton #1 *T*_1_ progenies . Results are expressed as the means ± standard errors (#2; *n* = 8) and (#6; *n* = 13): dark green fiber; (#10; *n* = 36) and (#17; *n* = 28): brown fiber; (Donor material ‘Zong 1282’; *n* = 47): brown fiber. (G) Fiber strength. (H) Fiber micronaire. (I) Fiber 25% span length. ‘a’, ‘b’, ‘c’ means with different letters are significantly different at *P* < 0.05. Means of three replicates ± SE, *, ** , ***-significant differences at *P* ≤ 0.05, 0.01 and 0.001 respectively.

Fiber quality and pigment color appear to be negatively correlated in naturally colored cotton (Upland cotton background) (Hua et al., 2007; Tan et al., 2013; Tu et al., 2014; Gong et al., 2014). For example, characteristics such as fiber length, micronaire, and fiber strength were lower in wild-type BCF and GCF than in WCF (Figs. 6A–6C). Fiber length and strength decreased as the intensity of the brown pigment increased. In wild-type green cotton, only the fiber strength decreased as the color darkened. The wild-type GCF was clearly shorter and weaker than the BCF in lightly colored cotton. The GCF was also considerably weaker than the intensely colored BCF (Figs. 6D–6F). An examination of the fiber quality of *GhCHI* transgenic offspring revealed that the fiber strength and micronaire of dark green fiber (T2–2 and –6) were the lowest in the same genetic background (Fig. 6G and 6H). Only BCF offspring had shorter fiber lengths than those of the parental genotype and dark green offspring (Fig. 6I).

Transcriptome and metabolome analyses revealed that flavonoid biosynthesis pathway genes were enriched during pigment synthesis. Silencing the *GhCHI-1* gene in a BCF line resulted in three fiber phenotypes, including GCF, among the RNAi lines’ offspring. Additionally, the overexpression of *Gh3GT* and *At3GT* genes demonstrated that the BCF turned green. Thus, the flavonoid biosynthetic pathway is responsible for BCF and GCF formation.

The GCF can be produced by interfering with the expression of *GhCHI-1*. Blocking the flavonoid biosynthetic pathway resulted in more substrates for the lignin biosynthetic pathway (Fig. S2). Because of the influence of transgene insertion sites, owing to regulation by epigenetic mechanisms, different gene suppression effects can be produced at different times.

Results from a previous study indicated that a defective *CHI* gene in the primary leaves of barley flavonoid mutants led to the accumulation of chalcone isosalipurposide (Reuber et al., 1997). Isosalipurposide synthesis likely influences the GCF phenotype (Fig. S2). The genomic sequence of the tetrahydroxychalcone 2′-glucosyltransferase gene is currently unknown. A multiple sequence alignment involving its (gi|156138818|) encoded amino acid sequence revealed a 56% identity to two *3GT* genes (Si3GT2L, gi|747104433| and Ns3GT2L, gi|698497982|). It also contained a sequence that was highly similar to the C-terminal region of *At3GT*, which encodes a fragment having an important glycosyltransferase activity ( Fig. S7). The relationship between isosalipurposide and green pigments in cotton fibers should be investigated. In naturally colored cotton, as the color darkened, the fiber quality decreased. Among all transgenic offspring, the quality of GCF was the lowest. As mentioned previously, wild-type GCF is of poorer quality (in terms of strength, length, and micronaire) than most conventional cottons. This may be related to the decreased *CHI* expression level at early stages, which affects fatty acid metabolism (Ngaki et al., 2012) and influences fiber quality.

Mostly WCF lines are the most important domesticated naturally colored cotton genotypes (Wendel, Flagel & Adams, 2012)*.* Lengthy domestication and directional selection processes resulted in long, strong, and fine white *G. hirsutum* fibers, as well as a high lint fiber yield per acre, and other economically important morphological traits.

The five stages of cotton fiber development consist of fiber initiation (0–3 DPA), primary cell wall synthesis and elongation (3–15 DPA), transition to secondary cell wall growth (15–20 DPA), secondary cell wall biosynthesis (20–40 DPA), and fiber maturation (40–50 DPA) (Haigler et al., 2012). Compared with domesticated cotton fiber, fibers of wild cotton species undergo a short primary cell wall synthesis and elongation stage (Applequist, Cronn & Wendel, 2001; Hovav et al., 2008; Hu et al., 2013). Transcriptome profiles confirmed this difference in fiber development of improved *vs*. wild cotton species (Yoo & Wendel, 2014). During the pigment formation stage, the flavonoid biosynthetic pathway is very active. This period is also critical for fiber elongation, and the products of the flavonoid biosynthetic pathway along with the combined activities of the auxin inhibitor naphthylphthalamic acid receptors and PIN-FORMED proteins may affect auxin transport (Jacobs & Rubery, 1988; Faulkner & Rubery, 1992; Mathesius et al., 1998; Murphy, Peer & Taiz, 2000; Peer et al., 2004). Therefore, the specific mechanisms affecting cotton development require further study.

It is unclear whether the flavonoid metabolic pathway beneficial effected cotton fiber development during the domestication process. It is possible that in addition to altering fiber color, fiber quality may be improved by regulating the timing of the expression of key flavonoid biosynthetic pathway genes.

Our research indicates that the formation of brown pigments in cotton fibers is controlled by the flavonoid biosynthetic pathway, and the formation of green pigments in cotton fibers may be controlled by the same pathway because the overexpression of *Gh3GT* and *At3GT* in BCF plants resulted in GCF. However, further research involving the transgenic offspring is required to identify flavonoid biosynthetic pathway genes that affect green pigment formation along with *Gh3GT*. Additionally, more comprehensive investigations of the relationships between flavonoid metabolism and fiber development are warranted.

## Conclusions

Taken these results together, our results indicate that the flavonoid biosynthetic pathway controls both brown and green pigmentation processes. Like natural colored fibers, the transgenic colored fibers were weaker and shorter than WCF. This study shows the potential of flavonoid pathway modifications to alter cotton fibers’ color and quality.

##  Supplemental Information

10.7717/peerj.4537/supp-1 Supplemental Information 1 Hierarchical clustering and quantitative real-time PCR validation of RNA-seq results. Phenylpropanoid pathway and genes involved in the biosynthetic pathways The differentially expressed anthocyanin genes were clustered using hierarchical clustering with Euclidean distance with complete linkage. The relative expression levels of these genes verified by quantitative real-time PCR were log2-transformed and presented using a color scale ranging from saturated blue for log ratios ≤ −1.5, to saturated red for log ratios ≥ 3.0. Each gene is represented by a row of colored boxes. The phenylpropanoid pathway includes the flavonoid/anthocyanin biosynthetic pathway (metabolites surrounded by a red box) and the lignin biosynthetic pathway (metabolites surrounded by a green box). PAL, phenylpropanoid (metabolic) pathway ; CHS, chalcone synthase; CHI, chalcone isomerase; THC2′GT, tetrahydroxychalcone- 2′-glucosyltransferase; F3H, flavanone 3-hydroxylase; F3′H, flavonoid 3′-hydroxylase; *F*3′5′H, flavonoid 3′, 5′-hydroxylase; DFR, dihydroflavonol 4-reductase; ANS, anthocyanidin synthase; 3GT, UDP-glucose:flavonoid-3-*O*-glucosyltransferase; 5GT, UDP-glucose:flavonoid-5-*O*-glucosyltransferase; MT, anthocyanin *O*-methyltransferase; AT, anthocyanin acyltransferase; CCR, cinnamoyl-CoA reductase; CAD, cinnamyl alcohol dehydrogenase; HCT, hydroxycinnamoyl CoA shikimate/quinate hydroxycinnamoyl transferase; CCoAOMT , caffeoyl-CoA *O*-methyltransferase .

10.7717/peerj.4537/supp-2 Table S1 The statistics of the 12 (samples: BCF, GCF, and WCF, numbered 5, 6, and 24, respectively at two time points, 0 and 12 DPA) library reads BCF, GCF, and WCF belong to wild type; numbered 5, 6, and 24 belong to transgenic type. There are two time points, 0 and 12 DPA.

10.7717/peerj.4537/supp-3 Table S2 List of real-time PCR primers in this study It mainly includes gene accession number, primer name, primer sequence, and the PCR product size.

10.7717/peerj.4537/supp-4 Table S3 Genes related to the phenylpropanoid (metabolic) pathway It mainly lists genes in the flavonoid metabolic pathway, including accession number and gene name.

10.7717/peerj.4537/supp-5 Table S4 Significantly differentially expressed genes (BCF and WCF) A comparison between BCF and WCF indicated that the significantly differentially expression genes were divided into nine clusters.

10.7717/peerj.4537/supp-6 Table S5 Significantly differentially expressed genes (GCF and WCF) A comparison between GCF and WCF indicated that the significantly differentially expression genes were divided into nine clusters.

10.7717/peerj.4537/supp-7 Table S6 Significantly differentially expressed genes (#5 and #24) A comparison between #5 and #24 indicated that the significantly differentially expression genes were divided into nine clusters.

10.7717/peerj.4537/supp-8 Table S7 Significantly differentially expressed genes (#6 and #24) A comparison between #6 and #24 indicated that the significantly differentially expression genes were divided into nine clusters.

10.7717/peerj.4537/supp-9 Table S8 List of pathways for significantly differentially metabolites The metabolomics of cotton fiber were analyzed by using the Q-TOF, and it was operated in both positive and negative ion modes. The pathway analyses of metabolites were performed with KEGG database sources to help identify the pathways that were most significantly altered.

## Additional Information and Declarations

### Data Availability

The following information was supplied regarding data availability: All sequencing data generated for this work have been deposited in the NCBI Gene Expression Omnibus (GEO) under accession number GSE76400.
