# Supplementary material for: Flavonoid biosynthesis controls fiber color in naturally colored cotton
Source: PeerJ. 2018 Apr 18;6:e4537. doi: 10.7717/peerj.4537 (PMC5910794; doi:10.7717/peerj.4537)
Supplement: Supplemental Information 1 — The differentially expressed anthocyanin genes were clustered using hierarchical clustering with Euclidean distance with complete linkage. The relative expression levels of these genes verified by quantitative real-time PCR were log2-transformed and presented using a color scale ranging from saturated blue for log ratios ≤ −1.5, to saturated red for log ratios ≥ 3.0. Each gene is represented by a row of colored boxes. The phenylpropanoid pathway includes the flavonoid/anthocyanin biosynthetic pathway (metabolites surrounded by a red box) and the lignin biosynthetic pathway (metabolites surrounded by a green box). PAL, phenylpropanoid (metabolic) pathway ; CHS, chalcone synthase; CHI, chalcone isomerase; THC2′GT, tetrahydroxychalcone- 2′-glucosyltransferase; F3H, flavanone 3-hydroxylase; F3′H, flavonoid 3′-hydroxylase; F3′5′H, flavonoid 3′, 5′-hydroxylase; DFR, dihydroflavonol 4-reductase; ANS, anthocyanidin synthase; 3GT, UDP-glucose:flavonoid-3-O-glucosyltransferase; 5GT, UDP-glucose:flavonoid-5-O-glucosyltransferase; MT, anthocyanin O-methyltransferase; AT, anthocyanin acyltransferase; CCR, cinnamoyl-CoA reductase; CAD, cinnamyl alcohol dehydrogenase; HCT, hydroxycinnamoyl CoA shikimate/quinate hydroxycinnamoyl transferase; CCoAOMT , caffeoyl-CoA O-methyltransferase . [file peerj-06-4537-s001.doc]

**Supplementary Information for**

**Flavonoid Biosynthesis Controls Fiber Color in Naturally Colored Cotton**

Hai-Feng Liu*, Cheng Luo, Wu Song, Hai-Tao Shen, Guoliang Li, Zhi-Gang He, Wen-Gang Chen, Yan-Yan Cao, Fang Huang, Shou-Wu Tang, Ping Hong, En-Feng Zhao, Jian-Bo Zhu, Da-Jun He, Shao-Ming Wang, Guang-Ying Huo, Hailiang Liu*

*Corresponding author. E-mail: cncclhf@163.com (L.H.F.); hailiang_1111@tongji.edu.cn (L.H.L.)


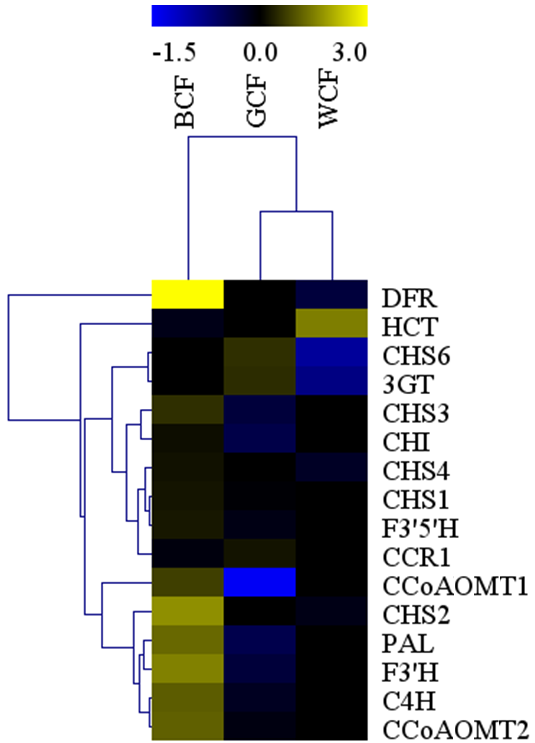


**Supplementary Fig. 1. Hierarchical clustering and quantitative real-time PCR validation of RNA-seq results.** The differentially expressed anthocyanin genes were clustered using hierarchical clustering with Euclidean distance with complete linkage. The relative expression levels of these genes verified by quantitative real-time PCR were log2-transformed and presented using a color scale ranging from saturated blue for log ratios ≤ −1.5, to saturated red for log ratios ≥ 3.0. Each gene is represented by a row of colored boxes.

**
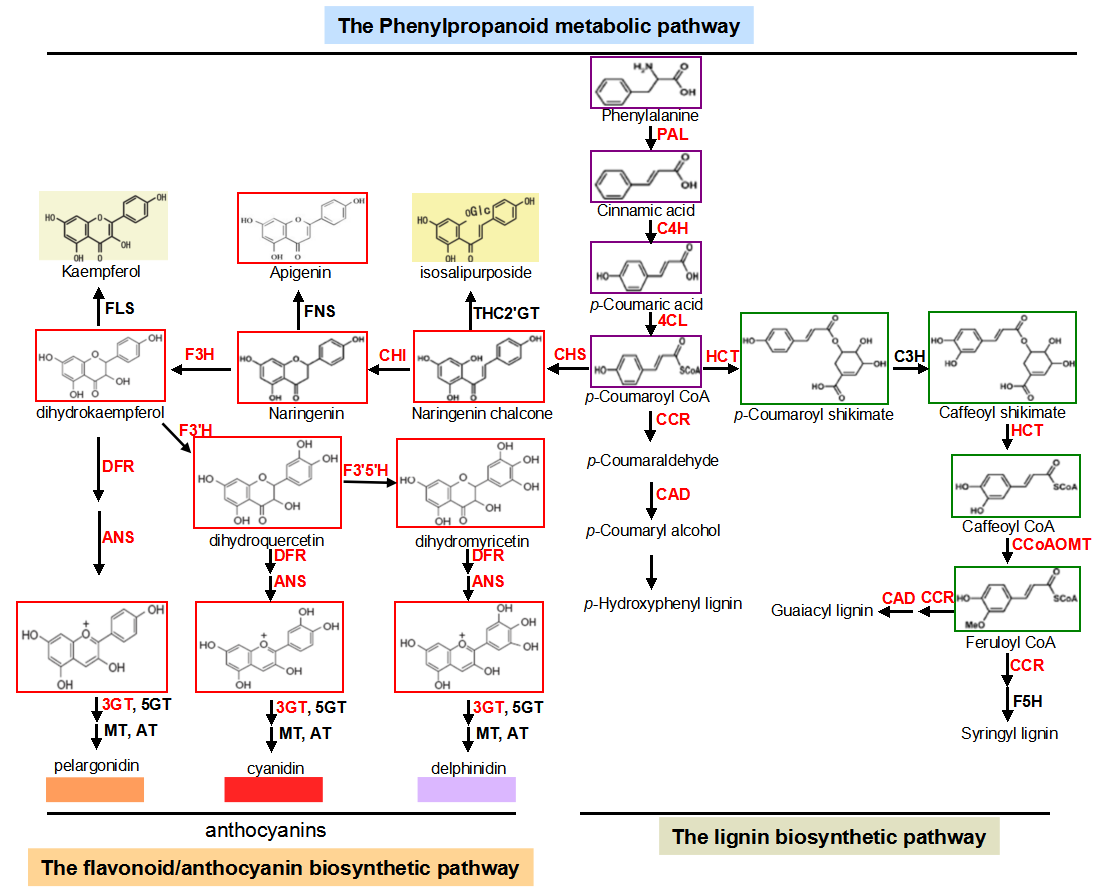
**

**Supplementary Fig. 2. Phenylpropanoid pathway and genes involved in the biosynthetic pathways.** The phenylpropanoid pathway includes the flavonoid/anthocyanin biosynthetic pathway (metabolites surrounded by a red box) and the lignin biosynthetic pathway (metabolites surrounded by a green box). PAL, phenylpropanoid (metabolic) pathway; CHS, chalcone synthase; CHI, chalcone isomerase; THC2′GT, tetrahydroxychalcone-2′-glucosyltransferase; F3H, flavanone 3-hydroxylase; F3′H, flavonoid 3′-hydroxylase; F3′5′H, flavonoid 3′,5′-hydroxylase; DFR, dihydroflavonol 4-reductase; ANS, anthocyanidin synthase; 3GT, UDP-glucose:flavonoid-3-*O*-glucosyltransferase; 5GT, UDP-glucose:flavonoid-5-*O*-glucosyltransferase; MT, anthocyanin *O*-methyltransferase; AT, anthocyanin acyltransferase; CCR, cinnamoyl-CoA reductase; CAD, cinnamyl alcohol dehydrogenase; HCT, hydroxycinnamoyl CoA shikimate/quinate hydroxycinnamoyl transferase;CCoAOMT, caffeoyl-CoA *O*-methyltransferase.

**
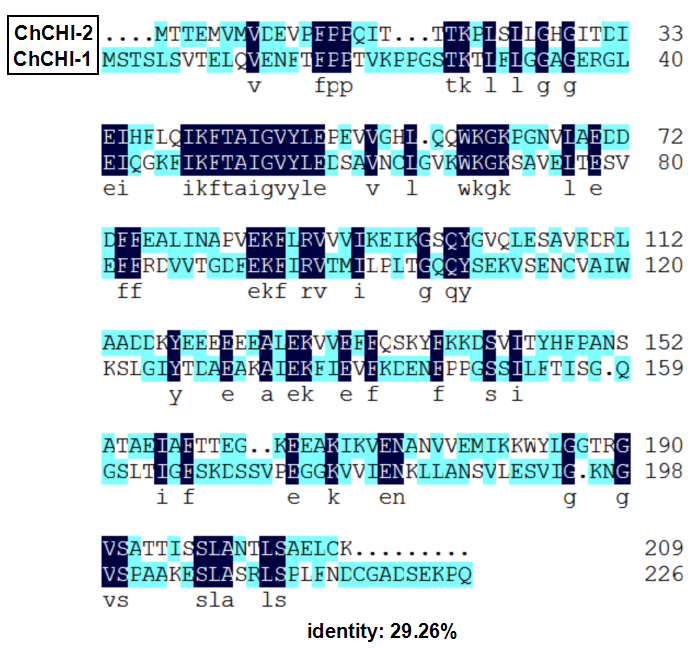
**

**Supplementary Fig. 3. Amino acid sequence similarities between GhCHI-1 and GhCHI-2.** Alignment of cotton (*Gossypium hirsutum*) GhCHI-1 and GhCHI-2. Shading indicates identity amino acid residues with identical residues highlighted in black. Letters below the aligned sequences represent conserved amino acids.


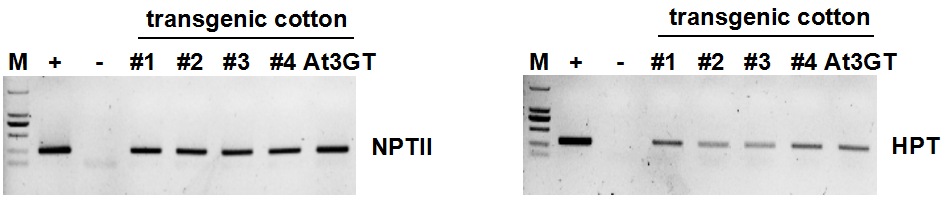


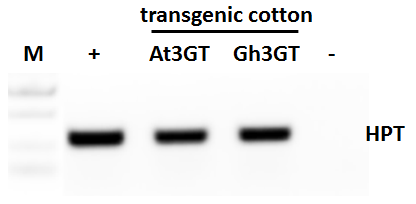


**Supplementary Fig. 4. PCR identification of transgenic cotton carrying *GhCHI-1* , *At3GT* and *Gh3GT*.** T_1_ generation plants were analyzed by PCR using primers specific for *NPTII* and *HPT*. The T_1_ generation consisted of four plantlets from 3,000 transformed brown cotton plants, and the T_0_ generation consisted of one *At3GT* and one *Gh3GT* transgenic plantlet from 774 and 1002 transformed green cotton plants, respectively. The transgenic plants were named #1, #2, #3, #4, At3GT and Gh3GT. M: DL2000 DNA marker.


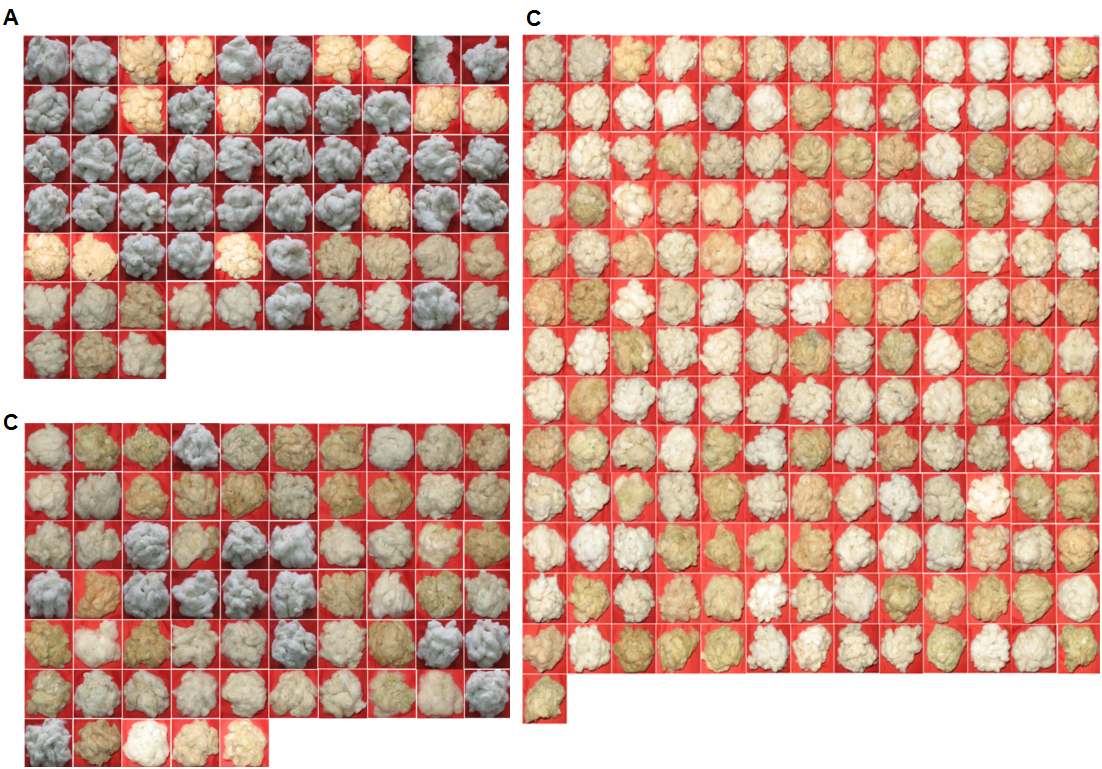


**Supplementary Fig. 5. Fiber color phenotypic analysis of transgenic cotton T_1_ progeny.**

(**a**) Transgenic cotton #2 T_1_ progeny (63 bolls). (**b**) Transgenic cotton #3 T_1_ progeny (65 bolls). (**c**) Transgenic cotton #4 T_1_ progeny (170 bolls).


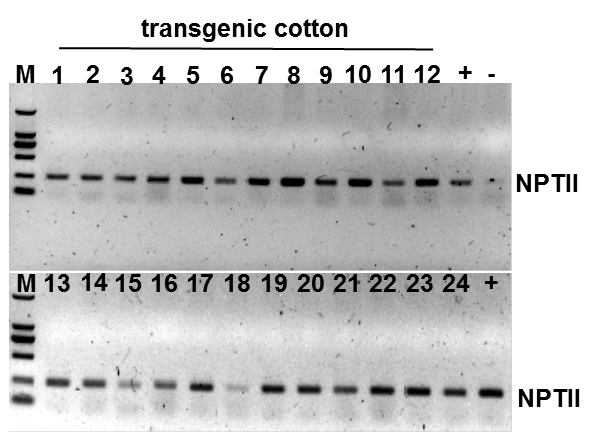


**Supplementary Fig. 6. PCR identification of T1 progeny of transgenic cotton #1 carrying *GhCHI-1*.**

T_1_ progeny of transgenic cotton #1 were analyzed by PCR using primers specific for *NPTII*. M: DL2000 DNA marker.

**Supplementary Fig.7. Alignment of CrTHC2′GT and 3GT proteins.**

CrTHC2′GT (*Catharanthus roseus*); Si3GT2L (*Sesamum indicum*); Ns3GT2L (*Nicotiana sylvestris*). Shading indicates identitical amino acid residues with 100, 75, and 50% conservation in black, pink, and turquoise, respectively. Letters below the aligned sequences represent conserved amino acids.
