# Supplementary material for: Flavonoid biosynthesis controls fiber color in naturally colored cotton
Source: PeerJ. 2018 Apr 18;6:e4537. doi: 10.7717/peerj.4537 (PMC5910794; doi:10.7717/peerj.4537)
Supplement: Table S1 — BCF, GCF, and WCF belong to wild type; numbered 5, 6, and 24 belong to transgenic type. There are two time points, 0 and 12 DPA. [file peerj-06-4537-s002.docx]

**Supplementary Table 1.**

**The statistics of the library reads**

| **Cotton type** | **Fiber color** | **Days post anthesis (DPA)** | **Raw reads** | **Clean reads** | **Mapped reads** |
| --- | --- | --- | --- | --- | --- |
| **Wild-type** | **BCF** | **0** | **11771495** | **10970680** | **6859639** |
|  |  | **12** | **11221271** | **11179449** | **7155492** |
|  | **GCF** | **0** | **10908353** | **10854565** | **7012968** |
|  |  | **12** | **11265624** | **11265624** | **6837262** |
|  | **WCF** | **0** | **10998125** | **10998125** | **7341128** |
|  |  | **12** | **12515205** | **12515205** | **7700367** |
| **Transgenic type** | **BCF** | **0** | **11417643** | **11417643** | **6581788** |
|  |  | **12** | **12697279** | **12697279** | **7875485** |
|  | **GCF** | **0** | **12328131** | **12328131** | **8251892** |
|  |  | **12** | **17609441** | **17609441** | **10557341** |
|  | **WCF** | **0** | **12848395** | **12848395** | **7651313** |
|  |  | **12** | **11237283** | **11237283** | **7125237** |

The stati窗体顶端

窗体底端
