# Supplementary material for: Flavonoid biosynthesis controls fiber color in naturally colored cotton
Source: PeerJ. 2018 Apr 18;6:e4537. doi: 10.7717/peerj.4537 (PMC5910794; doi:10.7717/peerj.4537)
Supplement: Table S3 — It mainly lists genes in the flavonoid metabolic pathway, including accession number and gene name. [file peerj-06-4537-s004.docx]

| **Metabolic pathway** | | **Gene accession number** | **Gene Name** |
| --- | --- | --- | --- |
| **phenylpropanoid metabolic pathway** | **lignin metabolic pathway** | **gi\|575502404\|** | **HCT (hydroxycinnamoyl CoA shikimate/quinate hydroxycinnamoyltransferase,** |
|  |  | **gi\|229368453\|** | **CCR (cinnamoyl-CoA reductase)** |
|  | **flavonoid metabolic pathway** | **gi\|289186761\|** | **3GT (UDP-glucose: flavonoid-3-**O-glucosyltransferase) |
|  |  | **gi\|153805695\|** | **CHS1 (chalcone synthase 1)** |
|  |  | **gi\|347466179\|** | **CHS2 (chalcone synthase 2)** |
|  |  | **gi\|347466183\|** | **CHS4 (chalcone synthase 4)** |
|  |  | **gi\|347466187\|** | **CHS6 (chalcone synthase 6)** |
|  |  | **gi\|121755800\|** | **CHI (chalcone isomerase)** |
|  |  | **gi\|310781378\|** | **F3'H** (flavonoid 3'-hydroxylase) |
|  |  | **gi\|197259949\|** | **F3'**5'H (flavonoid 3', 5'-hydroxylase) |
|  |  | **gi\|258489657\|** | **DFR (dihydroflavonol 4-reductase)** |

**Supplementary Table 3**.

**Genes related to the phenylpropanoid (metabolic) pathway**
